# A new exposure metric for traffic-related air pollution? An analysis of determinants of hopanes in settled indoor house dust

**DOI:** 10.1186/1476-069X-12-48

**Published:** 2013-06-19

**Authors:** Hind Sbihi, Jeffrey R Brook, Ryan W Allen, Jason H Curran, Sharon Dell, Piush Mandhane, James A Scott, Malcolm R Sears, Padmaja Subbarao, Timothy K Takaro, Stuart E Turvey, Amanda J Wheeler, Michael Brauer

**Affiliations:** 1 School of Population and Public Health, University of British Columbia, 2206 East Mall, Vancouver, BC, Canada V6T 1Z3; 2 Air Quality Research Division, Environment Canada, 4905 Dufferin Street, Toronto, Ontario, Canada M3H 5T4; 3 Faculty of Health Sciences, Simon Fraser University, 8888 University Drive, Burnaby, BC, Canada V5A 1S6; 4 Division of Respiratory Medicine, The Hospital for Sick Children, 555 University Avenue, Toronto, Ontario, Canada M5G 1X8; 5 Department of Pediatrics, Faculty of Medicine and Dentistry, University of Alberta, WC Mackenzie Health Sciences Centre, Edmonton, Alberta T6G 2R7, Canada; 6 Dalla Lana School of Public Health, University of Toronto, 155 College St, Toronto ON M5T 3M7, Canada; 7 Department of Medicine, Faculty of Health Sciences, McMaster University, 1280 Main St W, Hamilton ON L8S 4K1, Canada; 8 BC Children’s Hospital and Child & Family Research Institute, 950 West 28th Ave, Vancouver, BC, Canada V5Z 4H4; 9 Air Health Science Division, Health Canada, 269 Laurier Avenue West, Ottawa, Ontario, Canada K1A 0K9

**Keywords:** Air pollution, Dust, Exposure assessment, Hopanes, Land use regression, Traffic

## Abstract

**Background:**

Exposure to traffic-related air pollution (TRAP) can adversely impact health but epidemiologic studies are limited in their abilities to assess long-term exposures and incorporate variability in indoor pollutant infiltration.

**Methods:**

In order to examine settled house dust levels of hopanes, engine lubricating oil byproducts found in vehicle exhaust, as a novel TRAP exposure measure, dust samples were collected from 171 homes in five Canadian cities and analyzed by gas chromatography–mass spectrometry. To evaluate source contributions, the relative abundance of the highest concentration hopane monomer in house dust was compared to that in outdoor air. Geographic variables related to TRAP emissions and outdoor NO_2_ concentrations from city-specific TRAP land use regression (LUR) models were calculated at each georeferenced residence location and assessed as predictors of variability in dust hopanes.

**Results:**

Hopanes relative abundance in house dust and ambient air were significantly correlated (Pearson’s r=0.48, p<0.05), suggesting that dust hopanes likely result from traffic emissions. The proportion of variance in dust hopanes concentrations explained by LUR NO2 was less than 10% in Vancouver, Winnipeg and Toronto while the correlations in Edmonton and Windsor explained 20 to 40% of the variance. Modeling with household factors such as air conditioning and shoe removal along with geographic predictors related to TRAP generally increased the proportion of explained variability (10-80%) in measured indoor hopanes dust levels.

**Conclusions:**

Hopanes can consistently be detected in house dust and may be a useful tracer of TRAP exposure if determinants of their spatiotemporal variability are well-characterized, and when home-specific factors are considered.

## Background

Exposure to traffic-related air pollutants (TRAP) is associated with excess mortality [1,2]. The burden of air pollution from traffic on morbidity is also well documented with a variety of negative respiratory [3], cardiovascular [4] and reproductive effects [5] and lung cancer [6]. A recent comprehensive review concluded that there is sufficient evidence to infer a causal role for TRAP in the exacerbation of asthma in children and suggestive evidence of its role in the onset of asthma in children [7]. A number of pollutants (e.g. CO, NO_X_, and PM components) that are routinely measured at fixed regulatory monitoring sites have been used to represent exposure to TRAP. However, regulatory monitoring data cannot capture the fine-scale spatial pollutant gradients associated with vehicle emissions. Most of the recent epidemiological studies assessing TRAP have used methods with higher spatial resolution to provide individual-level exposure estimates. These methods generally estimate different surrogates of the traffic mixture (e.g. NO_2_, Black Carbon) derived from dispersion or land use regression (LUR) models [8]. Despite these advances in TRAP exposure assessment, none of the surrogate pollutants measured or modeled are specific to vehicle emissions.

In addition to the lack of specificity, these methods characterize ambient levels and do not consider indoor infiltration. Since individuals in North America spend an average of 87% of their time indoors [9,10] and many pollutants readily penetrate indoors, a significant proportion of total exposure to outdoor-generated pollutants occurs indoors. Quantifying the PM infiltration efficiency (F_inf_) in residences can help characterize indoor concentrations and reduce exposure misclassification [11] since F_inf_ can vary 2 to 10- fold between houses that have the same ambient concentrations [11-13].

Unfortunately, methods for estimating F_inf_ in residences require home-specific indoor and outdoor sampling, which makes estimating F_inf_ in large epidemiological studies virtually impossible. To overcome this limitation, prediction models of F_inf_ have been developed [12,13]. While these models have shown promise, they have generally been developed for individual cities using relatively small sample sizes and therefore may not be transferable to other locations.

Hence, current approaches to estimate individual TRAP exposures (LUR, dispersion model, geostatistical methods) have two consistent limitations: (i) TRAP surrogates are based on non-specific pollutant measures; (ii) modeled estimates predict concentrations outside the home while most exposure occurs indoors.

Settled house dust is a sink and repository for particle-bound material and semi-volatile organic compounds. Despite the variations that occur in sampling, dust measures have formed the backbone of epidemiological studies of multiple biological agents [14]. Indeed, house dust presents the advantage of providing one matrix for the evaluation of multiple agents which is a reasonable proxy for time-integrated exposure [15]. While the accumulation of house dust depends on several factors (e.g. infiltration efficiency, indoor and outdoor pollutant sources, cleaning practices, sampling surface), dust concentrations and loadings of pollutants show less variation over time than do indoor air concentrations, therefore, dust sampling is a particularly useful tool in studies of chronic exposures [16]. Measurement of airborne pollutants, for example of hopanes in PM_2.5_, is typically only conducted for short time intervals, use of air samples to assess chronic exposures would require longer sampling intervals or repeated measurements, features that are typically limited due to logistical (participant burden) or financial constraints. Using house dust as a marker for indoor inhalable hazards and infiltrated pollutants of outdoor origin (e.g. polycyclic aromatic hydrocarbons (PAHs) from vehicle exhaust) would represent a useful and readily available exposure assessment tool. A good tracer of TRAP in dust would be a chemical: 1) for which the major source is vehicle emissions; 2) for which emissions are correlated with other motor vehicles constituents; 3) that can be measured at low levels for reasonable cost; and 4) that can be measured with accuracy and stability.

One such group of tracers may be the hopanes, a class of organic compounds with 27 to 35 carbon atoms in a naphthenic structure [17]. Hopanes are not found in gasoline and diesel fuel because they are in the higher boiling fraction of petroleum, but are present in engine oil lubricants [18]. Hopanes are tracers of primary vehicular exhaust aerosols in ambient air [19], particularly on account of their relative stability and non-volatile nature in the atmosphere [20]. Schauer et al. showed that hopanes and steranes could be used to distinguish diesel and gasoline engine emissions from other combustion sources [21]. These relatively stable species can serve as unique tracers to determine the contribution of diesel and gasoline vehicles to particulate matter concentrations measured in outdoor air [3,22]. Measurement of hopanes in settled house dust may therefore be useful to estimate time-integrated exposure to TRAP, while also accounting for variability in infiltration. Our overall goals were to evaluate the potential utility of hopanes as TRAP exposure surrogates by determining (i) whether the hopane mixture in house dust had similar composition as that in outdoor air and (ii) the relationship between hopanes in settled house dust with predictors of TRAP spatial variability.

## Methods

We utilized indoor dust measurements from five Canadian cities spanning four provinces (from West to East: Vancouver (2.31 Million inhabitants), Edmonton (1.16 Million), Winnipeg (0.73 Million), Toronto (5.58 Million) and Windsor (0.32 Million) [23]) in order to ensure sufficient variability in hopane levels. Specifically, we conducted a city-level analysis where both city-specific and harmonized LUR variables across all cities were examined. These analyses also included covariates identified in housing characteristics surveys that were administered in the different studies used for this investigation.

### Population

Samples were collected in three separate studies, briefly described here, in which house dust was collected from inside homes of study participants:

1 the Canadian Healthy Infant Longitudinal Development (CHILD) study is a prospective longitudinal, birth-cohort study that has enrolled 3650 families from Vancouver, Edmonton, Winnipeg, and Toronto between 2009 and 2012. Homes that (i) underwent home assessment when the child was at an age of 3-months; (ii) completed the questionnaires on environmental factors, and (iii) had dust samples with sufficient dust mass for the analysis of several agents (endotoxins, β-glucans, and hopanes) were selected while ensuring balanced sample representation from the four CHILD cities. Thus, 120 homes analyzed for the suite of hopane monomers by December 2010 were included in this study;

2 The Toronto Child Health Evaluation Questionnaire (TCHEQ) with 1,500 subjects from a nested case–control study were randomly selected from a larger survey of 5,619 students who completed a screening survey for respiratory disease [24]. Within this nested study, a sub-sample of 50 homes were inspected in 2006/2007 and underwent measurement of indoor/outdoor concentrations of traffic related pollutants. From these, only 24 homes, included in this study, with sufficient dust mass for the analysis of allergens (Der *p*, Der *f*, Ergosterol and Glucans) were also analyzed for hopanes. [25];

3 During 2005/2006, Health Canada and the University of Windsor conducted a personal exposure study in Windsor [26] (the Windsor Ontario Exposure Assessment Study, WOEAS), in which 48 households were randomly recruited from the larger Windsor Children’s Respiratory Health Study [27] and where preference was given to spatially distributed households across Windsor. From these households, all homes with sufficient house dust mass were selected (n=27) to examine the hopanes levels in house dust.

### Hopanes

#### Dust samples: collection and analysis

House dust samples were collected from the living rooms in all the homes included in the study. Participants were asked not to vacuum during the week prior to the home visit. Sampling was conducted by trained technicians who were instructed to measure the sampling area, note the type of surface and collect a pre-determined amount of dust.

The WOEAS and TCHEQ sampling protocols were similar as technicians vacuumed a 4 m^2^ section of floor for a period of 4 minutes or until at least one gram of dust was collected and used high volume devices. In WOEAS, settled dust was collected using the High Volume Surface Sampling System (HVS3) vacuum [26], while TCHEQ used the Shop-Vac vacuum (Model: QAM70, 7.0 Amps), another high volume device, equipped with Dust Sampling Socks (X-Cell 100, Midwest Filtration, Cincinnati, OH, USA). In CHILD, house dust samples were collected using a standardized consumer model vacuum cleaner (Sanitaire, model S3686) fitted with a dust collection device designed especially for the CHILD study with the goal of increasing the collection efficiency without having to vacuum the entire area. This modified collector included slots for two nylon filter thimbles, thereby doubling the filtration surface and was constructed from machined aluminum, and outfitted with Teflon wheels to prevent marring of non-carpeted flooring, and to maintain the collection slot at a fixed distance from such floors. The sample was taken from a 2 m^2^ area by making seven passes of the nozzle over adjacent swaths of flooring. Only hopane concentrations in the family room were considered in the analysis since homes from WOEAS and TCHEQ studies did not provide samples from the bedroom and the bedroom samples in CHILD included a mixture of floor and bed samples.

All dust samples were sieved into <150 μm size fractions and reweighed for analysis. The sieved fractions were aliquoted and frozen at −80°C pending further analysis at the Environment Canada laboratory operated the Air Quality Research Division in Downsview, Ontario. Extraction in an isooctane solution was conducted with an ASE 200 (Accelerated Solvent Extractor) followed by solvent reduction using a Zymark TurboVap. Recovery standards were added to the dust/solvent matrix before extraction and blow down. A suite of organic compounds were quantified by tandem Gas chromatography–mass spectrometry, including eleven hopane monomers. The final dust-related metric for each of the individual hopanes and the sum of all 11 monomers was expressed as the concentrations per gram of sieved dust (ng/mg), thereby correcting for differences in the total amount of dust collected in each sample.

#### Outdoor hopane measurements

The composition of hopane mixtures, expressed as the abundance of the highest concentration monomer (17α(H), 21β(H)-Hopane) relative to the sum of the concentrations of all 11 measured monomers, was compared between available PM_2.5_ outdoor air samples in Vancouver, Edmonton, Toronto and Windsor with house dust samples for the same cities. In all cities, one 24-hr ambient PM_2.5_ sample was collected at Environment Canada national monitoring network (NAPS) sites [28] within the same week in the months of January, April, July and October 2010. The same suite of hopane monomers available in the dust samples were quantified at the NAPS Environment Canada Laboratory in Ottawa, Ontario, by thermal desorption gas chromatography mass spectrometry [29] from punches of archived pre-fired quartz filters. Dust and air samples were matched by city and season.

### Geographic predictor variables

Harmonized geographic data were derived to allow for pooled analysis of all dust hopane measurements from all five cities where samples were collected. We generated 30 variables in 5 categories that are often used in development of LUR models for TRAP [8,30]. Subcategories were generated to characterize the street network, land use, and population density within circular buffer sizes where the radius was set to represent close, medium and large geographical areas around each home where the house dust sampling was conducted (Table 1). Highways and major roads were defined by standard road classification categories (DMTI Spatial Inc., Markham, Ontario), with categories 1 (expressway), 2 (principal highway), and 3 (secondary highway) all considered highways (RD1), and category 4 as major roads (RD2). We also examined land use, elevation relative to sea level and the distance to the nearest features within the street network.

**Table 1:** Harmonized GIS data

**Category**	**Description**	**Sub-category**	**Buffer radii (m)**	**Source/type**
**(Number of variables)**				
Road Length	Total length of two road types	RD1 (Highways)	50, 100, 500, 1000	DMTI Road Network (Polyline)
(8)		RD2 (Major Roads)		
Land use	Total area of different land use types (ha)	COMM (commercial)	100, 500,1000	DMTI spatial data
(12)		OPEN		(polygon)
		PARK		
		INDUS (industrial)		
Distance to nearest feature	Distance to nearest road type (m)	Dist_RD1		
		Dist_RD2		
(6)				
	Distance to nearest land use type (m)	Dist_Comm		DMTI spatial data (polygon)
		Dist_Open		
		Dist_Park		
		Dist_Indus		
Population density	Density of the population (persons/hectare)	POPDENS	100, 1000, 2500	Block level census data (point file)
(3)				
Geographic position	Elevation (m)	ELEV		Geobase DEM (raster)

All variables in each category were derived from a single spatial dataset in vector format. Input files for the Road Length and Land Use were taken from the 2006 DMTI Spatial (Markham, Ontario) data files. Population Density categories were generated from the 2006 census distributed by Statistics Canada and converted into point files at the block level. Digital Elevation Data was obtained from GeoBase in raster format at the municipal level. All input files were manipulated in ArcGIS 10 (ESRI, Redlands, CA) to produce variable layers in raster format at 10 m resolution, except for the digital elevation model where the finest available resolution was 30 m. From the latter data, relative elevation was defined as the mean centered city-specific elevation.

We also extracted city-specific variables that had previously been extracted and used in the development of LUR models for NO_2_ in each of the cities [31-34] (see Additional file 1). Since these LUR variables had been used to explain variability in outdoor NO_2_ in these cities we therefore expected that they would explain variability in dust hopanes concentrations.

### Questionnaires

We also included data from questionnaires delivered in each of the indoor measurement studies on housing characteristics and lifestyle factors, which may be related to indoor hopane variability and/or infiltration (Table 2).

**Table 2:** Descriptive summary of questions found (as shown with a check mark) in the questionnaires delivered during home visits, recoded for analysis in the pooled investigation of hopanes in dust and land use determinants of traffic pollution

**Question type**	**CHILD**	**TCHEQ**	**WOEAS**
Emissions sources within 100 m of the home	√	×	×
Factory	2%		
Gas station	11.3%		
Parking	15.6%		
Construction site	23.5%		
Shoe removal	√		
Yes	94%	×	×
No	6%		
Type of floor	√	√	√
mixed	4%	83%	4%
smooth	21%	17%	36%
carpets	75%		60%
Cleaning frequency	√	√	√
Rarely	14%	4%	56%
Moderately	80%	71%	44%
Frequently	6%	25%	
Window usage/type	√	√	√
Usually open/sheer	15%	37.5%	33.3%
Covered with blinds/curtains	42.5%	14.8%	63%
Sealed	34%	14.8%	3.7%
Opened daytime/ closed night Other	2%	14.8%	0%
Garages	√	√	√
Yes	46%	17%	52%
No	54%	83%	48%
Type of house	√	√	√
single	64%	100%	100%
multifamily	36%		
Air conditioning	√	√	√
Yes	40%	100%	81%
No	60%		11%
Frequency of AC use	√	√	√
Frequently	21%	42%	15%
Sometimes	19.5%	46%	7%
Don’t know	59.5%	12%	
Never	0		78%

For homes that were part of the CHILD study, home information was gathered from both a questionnaire completed by the parents and the home inspection conducted by research technicians. For homes that were part of TCHEQ, a large amount of housing characteristics data were also available from a questionnaire that was administered at study baseline (633 questions). Finally, from the WOEAS homes for which information on a wide range of housing characteristics and time-activity patterns was collected twice, we used the baseline questionnaire. The questionnaires included questions that were unique to each cohort as well as other questions common across all studies (see Table 2), which were recorded to generate a set of harmonized data. Harmonized variables included data related to the season (defined using heating degree day) based on the date when samples were collected, the type of floor (recoded as smooth for hard wood, vinyl and other smooth surfaces, carpets for rugs and carpets, and mixed for samples collected from both smooth and carpet surfaces), the type of household (single or multifamily), the presence or absence of a garage, the type of garage (attached or detached), the presence of air conditioning (central or in a wall or portable unit), the frequency of use of air conditioning (recoded as never, sometimes and frequently), the cleaning frequency (recoded as rarely, sometimes and frequently), and the usage of windows (recoded and grouped from different questions in the CHILD questionnaire) coded into 5 categories: usually open/sheer; covered with blinds or curtains; sealed; open daytime/covered nighttime; other.

### Statistical analysis

We first analyzed the association between the mixture of hopanes in outdoor air and indoor dust by comparing the relative abundance of the most abundant monomer. Specifically, we calculated the ratio of the concentration of 17α(H), 21β(H)-Hopane to the total concentration of the eleven monomers. We then compared this relative abundance between the outdoor air and indoor dust samples in each city, and examined this association after accounting for temperature and evaluating multicollinearity between predictors (assessed by the variance inflation factor) in linear regression models

After examining the distribution of hopane concentrations in a pooled analysis of dust samples from all cities (hereafter “pooled analysis”) and separately within each city (“city-specific analysis”), we applied a log transformation to the total hopane concentration (i.e. sum of the 11 monomers) distribution across all cities and within each city. Prior to examining the association between total hopane concentrations and GIS variables in the pooled analysis, we fit a random effects model with a random intercept at the city level to assess the between- and within-city variability. Both in the pooled and city-specific analyses, questions on lifestyle factors and housing characteristics were examined in bivariate analysis as potential confounders or effect modifiers for the hopane – geographic predictor relationships.

The same model building approach described by Henderson et al. [33] to generate physically meaningful predictive models was adopted and consisted of the following steps: (1) Rank all variables by the absolute strength of their correlation with the hopane concentration; (2) Within each sub-category (e.g. all buffer sizes for highway lengths), keep only the highest-ranking; (3) to avoid collinearity examine the correlations between all GIS predictors retained from step 2 as well as questionnaire variables using 0.6 as a cut-off value ; (4) enter all remaining variables into a stepwise linear regression; (5) remove from the available pool any variables that have insignificant t-statistics and variables that show a direction of effect opposite of a priori hypotheses. These five steps follow previous LUR models [8] and the general approaches often used in determinants of exposure modeling, for example in assessment of occupational exposures [35]**.** Steps 4 and 5 were repeated until a parsimonious final model that best explained the variations in indoor dust hopanes levels was obtained.

The study methodology was reviewed and approved by both the University of British Columbia Behavioral Research Ethics Board (ethics certificate no-H11-03231) and the Clinical Research Ethics Board (H07-03120).

## Results

### Outdoor vs. indoor hopane concentrations comparison

**Figure 1:**
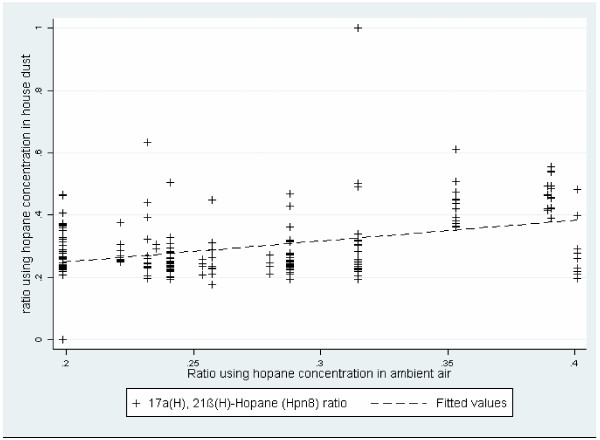
Association between outdoor air and house dust hopane major monomer (17α(H), 21β(H)-Hopane) relative abundance.

All samples were above the GC/MS limit of detection (LOD). In all cities the monomer 17α(H), 21β(H)-Hopane was consistently detected and showed the highest abundance in the suite of analyzed compounds, therefore the comparison of outdoor and indoor hopane ratios was performed using this monomer relative to the sum of all monomers. The sampling from the air monitoring stations was conducted in 2010 at fixed time points which result in concentrations from air samples with a discrete distribution (Figure 1) compared with the house dust samples which were collected throughout the year. In air, the range of the 17α(H), 21β(H)-Hopane relative abundance (0.2 to 0.4) generally corresponded to the same relative abundance in house dust. The correlation of the 17α(H), 21β(H)-Hopane relative abundance in outdoor air and house dust was moderately strong, yet significant (r=0.48, p<0.05).

After excluding an outlier sample (see Figure 1 data point near zero where the ratio in 17α(H), 21β(H)-Hopane was depleted due to a very low concentration in all monomers), we also examined the relation between the outdoor and the indoor relative abundance in linear regression accounting for the effect of season, and found a stronger statistically significant relationship (slope=0.92, t=6.1) compared to the association without adjustment for season (slope=0.72, t=5.9). The correlation was still significant when the outlier was included. The effect of season was stronger during the spring and summer (r>0.5) than during the fall and winter (r<0.5).

### Pooled and city-specific results

Hopane levels in individual homes varied from a low of 0.4 ng/mg of dust in a Toronto home to a high of 41.8 ng/mg of dust in a Vancouver sample, after excluding an outlier in Toronto from the CHILD study where the concentration was 160.3 ng/mg (more than 29 times higher than the median); for this home we examined the land use characteristics, the road network, and potential outdoor sources as indicated in the questionnaire and did not find any difference that would explain such a high concentration. Analyses were thus run with and without this sample (Table 3).

**Table 3:** City-specific determinants of hopane concentrations in house settled dust

**City**	**Final model with regression coefficients**	**Partial R**^**2**^	**Model Adj.R**^**2**^
Edmonton	log (hopanes) = 3 – 0.13 cleaning frequency	0.78	0.80
	- 1.5 Smooth Flooring	0.78	
	−0.15 Air Conditioning	0.35	
Toronto	Log (hopanes) = 2.69 -1.03 Smooth Flooring	0.29	0.45
	−0.008 Elevation	0.13	
	+ 0.88 Attached Garage −0.66 Detached Garage	0.13%^*^	
Windsor^**^	log (hopanes) = 5.6 + 0.5 elevation	0.36	0.39
	+ 0.17 RD1_100	0.13	
Winnipeg	log (hopanes) = 1.45 – 0.057Heating degree days	0.17	0.33
	– 1.33 multifamily house	0.16	
Vancouver	log (hopanes) = 1.9 – 0.09 Heating Degree Days	0.09	0.10
	– 0.07 Shoe removal	0.07	

^*^ The Garage variable has three categories: No garage, Attached garage and Detached garage.

^**^ In Windsor, elevation and distance to the Ambassador Bridge were strongly and significantly correlated. An alternative model with Distance to Ambassador Bridge yielded similar results, yet with smaller R^2^

Windsor had the lowest overall indoor hopane levels with a mean level of 5.8 ng/mg (GM= 5.1 ng/mg, GSD =1.8) while the sample of homes in Vancouver showed the highest mean concentration of 9.3 ng/mg (GM= 6 ng/mg, GSD= 2.9) when the high (Toronto) outlier was excluded (if the Toronto outlier was retained, then Toronto was ranked first with an AM=9.9 ng/mg).

#### Pooled analysis

After fitting a null random effect model, the intraclass correlation of 0.007 indicated that city clustering would not contribute to explaining the variability in total hopane concentrations. We therefore built a model without using a city-specific random intercept in the regression analysis and all samples were treated as independent.

From the harmonized questions, only relative elevation and heating degree days at the time of dust collection showed a statistically significant relationship with hopane concentrations. Distance to highway (DIST_RD1) had a statistically significant association with hopane concentrations, but its direction of effect was opposite to a priori expectations and was therefore excluded from the model. The final model with relative elevation and heating degree days as predictors explained only 6% of the total variability in the hopanes concentration. Including distance to highway did not appreciably improve the amount of explained variability (adjusted R^2^ =0.08). Excluding the high outlier home in Toronto led to a model with the addition of the presence of an AC unit in the home along with the same predictors as above, but with less overall variability explained (adjusted R^2^= 0.04).

#### City-Specific modeling results

Given the availability of LUR models for predicting NO_2_ in each study area, we extracted the NO_2_ concentration at the geocoded participants’ home addresses and examined the correlation of hopane concentrations in house dust with city-specific LUR NO_2_ estimates in each city. Results (Table 4) indicated no statistically significant associations except in Windsor (r=0.44, p<0.05) and Edmonton (r=0.58, p<0.05).

**Table 4:** **Geometric Mean (GM) and Geometric Standard Deviation (GSD) of total Hopanes concentrations in the study population, by room, by home and correlation a with city-specific modeled NO**_**2**_

**City**	**Homes**	**Hopanes concentration (ng/mg)**						**Pearson correlation**
	**N (number of homes)**	**Bedroom**		**Family room**		**Average**		**Between NO**_**2 **_**and family room**
		**n**	**GM (GSD)**	**n**	**GM (GSM)**	**N**	**GM (GSD)**	**r (p-value)**
Winnipeg (CHILD)	26	23	4.9 (2.1)	21	5.8 (2.1)	40	5.3 (2.3)	0.04 (n.s.)
Edmonton (CHILD)	15	12	4.7 (2.7)	14	4.1 (2.0)	26	4.5 (2.3)	0.58 (0.03)
Vancouver (CHILD)	65	56	7.4 (2.2)	54	6 (2.9)	90	6.7 (2.6)	-0.12 (n.s.)
Toronto (CHILD)	14	13	5.9 (1.9)	12	7.7 (2.9)	22	6.6(2.3)	0.02 (n.s.)
Windsor (WOAES)	27	NA		27	5.1 (1.8)	NA		0.44(0.02)
Toronto (TCHEQ)	24	NA		24	4 (2.5)	NA		0.18 (n.s.)

Abbreviations: *n* total number of samples, *n.s.* not statistically significant association.

Leveraging the availability of city-specific LUR models, we further examined separately for each city the association between hopane concentrations and the variables that were used both in the city-specific LUR models describing the NO_2_ levels (see Additional file 1) and those that we generated for the pooled analysis (Table 2). The amount of variability explained in each city varied from 10% in Vancouver to 80% in Edmonton (Table 3).

Overall, in each city the determinants of indoor dust hopanes were predominantly related to home-specific factors (cleaning, use of AC, shoe removal) and meteorology, except for Windsor where the final model included the length of major roads in a 100m buffer (Table 3). In Toronto, the spatial variability provided by the TCHEQ samples was very limited as all homes were within a restricted geographic area within the city. Hence, an additional sub-analysis was run for Toronto with only the CHILD homes included. This model (not shown) did retain GIS variables (open area within 1000 m buffer and elevation) as well as variables related to other possible sources of hopane emissions (garage type, presence of a construction site within 100 m) and finally home-specific factors (i.e. the type of floor surface) and explained 86% of the overall variability in indoor dust hopanes. After excluding the house with the outlier concentration value, however, the final model, with an R^2^ = 0.3, had exactly the same predictors as those shown in Table 3 where samples from both the TCHEQ and CHILD study homes in Toronto were included.

While the association of hopanes indoors in relation to GIS variables typically used as surrogates for TRAP was only modeled for samples collected in living rooms, Table 4 shows the concentration in each city by room type and the number of homes (from the CHILD study) where two rooms were sampled. In CHILD, participating households provided dust samples from the living room as well as a second composite sample from subject child’s mattress and adjacent flooring. The ranking by decile showed that the hopane concentration in the living rooms was significantly greater than that found in the bedrooms.

## Discussion

Assessing indoor levels of TRAP through the collection and analysis of settled house dust is a new area of study and has the potential to reduce the misclassification and increase the specificity of exposure. In this investigation, we compared hopanes in dust and ambient air and with GIS-derived land use variables. This is the first investigation of hopanes collected in house settled dust. The availability of contemporaneous cohort studies (CHILD, TCHEQ and WOEAS) offered a unique opportunity to gather a sample of 171 homes where dust was collected using similar protocols in 151 living rooms and where hopanes were analyzed by GC/MS at the same laboratory using a standardized protocol. Samples were collected from different settings ranging from highly urban locations such as Toronto to smaller and less densely populated cities such as Winnipeg, while also including major transit hubs such as Windsor, the site of a major Canadian-American truck border. Furthermore, all the cities had previously developed LUR models which reasonably predicted traffic related NO_2_ spatial variability (from 66% in Vancouver to 81% in Edmonton [31,33].Still, these homes represent only a small fraction of the total homes in each city and even of the homes included in each of the studies. Further, different numbers of homes were included in the different cities. We are therefore unable to make conclusions regarding the representativeness of the measured hopanes levels and instead focused on the variability within and between cities and the extent to which this variability could be explained by various potential determinants.

We demonstrated that hopanes can be consistently detected in house dust samples regardless of the type of city and the dust collection location. In addition, after controlling for heating degree days and its impact on infiltration, the major hopane monomer relative abundance in house dust and outdoor air samples were significantly correlated (r = 0.48), suggesting similar hopane sources in the two samples, but there remains substantial unexplained variability in indoor levels. This comparison had relatively good external validity given that the ambient monitoring sites were located to capture urban background concentrations rather than hot spots and since samples were collected in and matched for all seasons. This correlation was stronger in the summer compared to the winter, suggesting an impact of infiltration as windows are more likely to be opened on warmer days. Since hopanes in house dust accumulate over relatively longer periods of time compared with hopanes in air samples and may have undergone many changes and cycles in temperature, it is likely that the seasonal effect shown in the literature [36-38] may not hold in this context. In addition, dust sampling, which often is a readily available matrix for sampling multiple agents in epidemiological studies, including hopanes as demonstrated in this study, does not represent similar constraints (e.g. logistics) as those imposed by particle infiltration measurements.

Examining associations between hopane concentrations and geographic predictors in a pooled analysis indicated that only a small degree of variability in hopane concentrations in dust was explained by the final model. Further, in this analysis, higher levels of road variables were linked to lower levels of hopanes. Despite the advantages of pooling data from different cohorts, this effort was hindered by the absence of consistency in the supplementary data collected via questionnaires since each study used its own set of questions. While we inspected each question and the research technicians’ notes for each sample of house dust collected in order to generate harmonized variables that could affect the hopane concentration in house dust, recoding variables may have resulted in a loss of specificity.

Unlike the pooled analysis, the city-specific analysis provided more insight into the utility of hopanes as possible markers for TRAP as a moderate to large amount of variability in the total hopane concentration in house dust was explained in each model. This analysis, however, was hindered by the lack of consistency between cities in terms of main predictors of indoor hopane concentrations.

We examined potential modifiers that could alter the relationship between LUR variables and hopanes in dust for each city separately. In addition to geographic surrogates of TRAP, all cities had at least one predictor of hopane concentration related to the indoor environment or home construction. Thus, the variability in settled dust hopane concentrations appears to be a function of a mix of parameters that are not exclusively related to traffic emissions.

For example, indoor hopanes in settled dust may also result from coarse PM being tracked indoors. A recent analysis of indoor PAHs indicated the potential importance of this pathway even after adjusting for carpeting, frequency of vacuuming and indoor burning [39]. We examined the association in the city-specific analysis for all CHILD participating homes between shoe removal habits and hopane concentrations. We found that only Vancouver samples were correlated with shoe removal habits in the expected direction. Collection of supplementary field data remains a crucial component for assessing the utility of hopanes in house dust since tracked dust seems to contribute to hopanes concentration in house dust. This information was only available in the CHILD homes, and could therefore not be assessed in the pooled analysis.

In our investigation we made a critical assumption that hopanes have few sources beyond engine oil lubricants as we were not able to find information on indoor hopane sources in the literature. Since hopanes are widespread in recent and ancient sediments, they are constituents of all mineral oil or petroleum-based lubricants and it is therefore possible that unaccounted for indoor sources were present.

House dust remains an attractive metric for exposure assessment because it offers a matrix for multiple indoor contaminants, both biological and chemical and both indoor and outdoor in origin, and can be stored for long time periods, thus providing the opportunity to examine additional research questions when necessary. The utility of hopanes in house dust as an indicator of infiltrated TRAP is limited in the absence of better understanding of its deposition and stability in house dust. House dust is heterogeneous matrix with a complex history in each home as it accumulates contributions from multiple sources including not only fresh emissions of combustion-related particles but also road dust which also contains hopanes. The mode of accumulation also contributes to the variability of vacuum dust. Several factors that may vary among study participants can affect the concentrations of hopanes: cleaning practices and sampling surfaces (carpeted vs. non-carpeted) play a role in the amount of chemicals that deposit inside the homes as shown in the city-specific analysis. In addition, the metric of exposure for hopanes still lacks consensus as hopanes can be measured in terms of loading (concentration normalized by surface area sampled) or expressed as the more traditional approach of normalized concentration to mass of dust collected. Differences in the choice of metric would relate mostly to cleaning practices, which we have tried to account for in our investigation. Future investigations of other species, such as PAHs, on their own or in combination with hopanes, may offer additional insight into the utility of settled house dust as a surrogate for TRAP exposure.

In our study, we compiled the information about presence and frequency of use of air conditioning as this has been shown to be an important predictor of PM infiltration [40], but we found limited explanatory power in both pooled and city-specific analysis. PM infiltration varies with particle size, with a maximum infiltration efficiency for diameters of approximately 0.2-0.3 μm [41], while the size distribution of hopanes ranges between 0.7 and 3.3 μm [42] which would imply that hopane infiltration efficiency may be low and might therefore explain variability in the outdoor/indoor correlation [37]. We could expect that in presence of higher concentrations of hopanes in ambient air (i.e. better ability to detect hopane monomers), the analysis of relative abundance in ambient and indoor hopane would have shown less unexplained variability.

## Conclusions

Our results indicate that indoor dust hopane concentrations depend on both outdoor TRAP and on a variety of home-specific variables such as cleaning, floor type, and presence of AC. This conclusion is supported by our analysis of the relative variation explained by LUR NO_2_ compared to home-specific factors as we also found that in some cities a correlation between hopanes and LUR NO_2_ is only revealed when accounting for variation due to such home-specific factors.

We examined the utility of measurements of hopanes in house dust as exposure indicators for infiltrated, time-integrated, traffic-related pollutants. When combined with behavioral factors retrieved from questionnaires, and geographic determinants, hopanes in house dust may have the potential to be used as surrogates for infiltrated TRAP. Further characterization of the determinants of hopanes in house dust may result in an improved exposure measure for epidemiologic studies to more precisely analyze relationships between TRAP and chronic health effects.

## Consent

Written informed consent was obtained from the study participant’s guardian/parent for the use of personal information kept confidential and only used for scientific objectives.

## Abbreviations

CHILD: Canadian Healthy Infant Longitudinal Development; GIS: Geographic Information Systems; GM: Geometric Mean; GSD: Geometric Standard Deviation; LUR: Land Use Regressions; TCHEQ: Toronto Child Health Evaluation Questionnaire; TRAP: Traffic-Related Air Pollution; WOEAS: Windsor Ontario Exposure Assessment Study.

## Competing interests

All authors declare no competing financial interests.

## Authors’ contributions

HS formulated the research question, gathered spatial data and extracted questionnaire data, conducted the statistical analysis and led the writing of the manuscriptMB guided the study design, provided spatial data for Vancouver, helped formulate the study questions, gave critical input in data conditioning for the analysis and revised the manuscript for its intellectual content. RA provided the spatial data for Edmonton and Winnipeg and guided the data harmonizing across different spatial databases. JB gathered the ambient air samples from the national air monitoring stations and oversaw the chemical analysis of hopanes samples in Environmental Canada Laboratory. JC performed a preliminary analysis examining personal hopanes in dust and air samples. JB, JS, TK, RA, and MB are part of the Exposure Working Group for the CHILD study where all questionnaires used in this study were designed. This group, along with the site leaders and CHILD PI oversaw the collection and initial processing of CHILD dust samples. ST, PM, PS are site leaders for the CHILD study, they are responsible for the coordination and training of research technicians and data collection. MS is the Principal Investigator of the CHILD study and has revised the manuscript drafts. SD is the Principal Investigator of the TCHEQ study and revised the manuscript. AW has led the WOEAS, conceived of this study and participated in preparing the spatial data for Windsor, she has given critical input to the manuscript drafts. All authors have contributed to this manuscript and given approval to the final version.

## Supplementary Material

Additional file 1 City-specific GIS variables and buffer sizes extracted from LUR model surfaces. Click here for file
