# Supplementary material for: A new exposure metric for traffic-related air pollution? An analysis of determinants of hopanes in settled indoor house dust
Source: Environ Health. 2013 Jun 19;12:48. doi: 10.1186/1476-069X-12-48 (PMC3711892; doi:10.1186/1476-069X-12-48)
Supplement: Additional file 1 — City-specific GIS variables and buffer sizes extracted from LUR model surfaces. [file 1476-069X-12-48-S1.docx]

# Additional file 1

City-specific GIS variables and buffer sizes extracted from LUR model surfaces.

| **City/ Cohort** | **Road metrics (m)** | **Traffic density** | **Other** |
| --- | --- | --- | --- |
| Edmonton CHILD | Length of all roads  (100,250, 500, 1000m) |  | Distance to city center (m)  Water area |
| Winnipeg CHILD |  |  | Y coordinate centered at city center. |
| Vancouver CHILD | Length of Truck roads  (100,250, 750, 1000m) | Automobile density  Truck density  (both in buffer with radius size: 100, 250, 750, 1000m) |  |
| Toronto TCHEQ | Distance to nearest expressway   Length of expressways  (100,500, 1000, 2000m) |  |  |
| Windsor WOEAS | Distance to nearest local road Distance to nearest class 1, 2 collector  Distance to nearest class 1, 2 arterial  Distance to nearest highway Distance to nearest scenic drive |  | Distance to Ambassador bridge (m) Distance to Windsor/Detroit Tunnel (m) |
